# Real-Time Detection of Sporadic Meteors in the Intensified TV Imaging Systems

**DOI:** 10.3390/s18010077

**Published:** 2017-12-29

**Authors:** Stanislav Vítek, Maria Nasyrova

**Affiliations:** Faculty of Electrical Engineering, Czech Technical University in Prague, Technicka 2, 166 27 Prague, Czech Republic; nasyrmar@fel.cvut.cz

**Keywords:** sporadic meteor, real-time detection, image intensifier, meteor automatic imager and analyzer, graphical processing unit

## Abstract

The automatic observation of the night sky through wide-angle video systems with the aim of detecting meteor and fireballs is currently among routine astronomical observations. The observation is usually done in multi-station or network mode, so it is possible to estimate the direction and the speed of the body flight. The high velocity of the meteorite flying through the atmosphere determines the important features of the camera systems, namely the high frame rate. Thanks to high frame rates, such imaging systems produce a large amount of data, of which only a small fragment has scientific potential. This paper focuses on methods for the real-time detection of fast moving objects in the video sequences recorded by intensified TV systems with frame rates of about 60 frames per second. The goal of our effort is to remove all unnecessary data during the daytime and make free hard-drive capacity for the next observation. The processing of data from the MAIA (Meteor Automatic Imager and Analyzer) system is demonstrated in the paper.

## 1. Introduction

Meteors are streaks of light appearing in the sky when meteoroids ablate in the Earth’s atmosphere. Observation of meteors is a cost-effective way to understand the distribution of material in our solar system. Meteor observations are typically performed using radar [1], passive radio detectors [2], all-sky photographic [3] and CCD (charge coupled device) cameras [4], digital video cameras [5] or television (TV) cameras optionally equipped with an image intensifier. While the radio-based detection methods can be performed during the daytime, thus being suitable for estimation of total meteor activity, camera-based methods are limited to night time. Regardless of this limitation, camera-based observations allow building the light curve (i.e., the time-dependent fluctuations of light emitted by a meteor), which may contain information about the mass and structure of the original particle or parent object: comets [6] and asteroids [7]. Wide-band observation with a suitably-designed bank of photometric filters additionally allows obtaining information about the chemical composition of the meteoroid [8,9]. Although camera-based systems are more common, combinations of multiple ways of observations are also used [10]. All-sky cameras with a huge spatial resolution and long exposure times are great for detecting intense light phenomena, like bolides or fireballs. However, for the calculation of atmospheric trajectory, it is necessary to observe meteors simultaneously from at least two different places, optionally with high temporal resolution. Moreover, a higher frame rate brings more data for the modeling of the meteoroid structure [11].

Meteor observation with two or more camera systems is currently a standard technique for the measurement of meteoroid trajectories. There are networks of different scales and technology: the Spanish Meteor Network (SPMN) [12] has about 25 video and CCD stations; Cameras for Allsky Meteor Surveillance (CAMS) [13] operates more than 60 narrow-field cameras at five locations in the United States (three in California, one in Florida and also on the Mid-Atlantic coast). The concept was later applied by amateur astronomers in the Netherlands and Belgium [14]. The Croatian Meteor Network (CMN) [15] employs more than 30 cameras. The Polish Fireball Network (PFN) consists of 36 continuously-active stations with 57 sensitive analogue video cameras and 7 high-resolution digital cameras [16]. The Canadian Automated Meteor Observatory (CAMO) uses four CCD cameras running at 80 frames per second (fps) and coupled to 18-mm image intensifiers [17]. The Desert Fireball Network (DFA) currently covers one third of Australia (approximately 2.5 million km${}^{2}$) with the use of 49 digital single reflex camera (DSLR)-based stations with nominal spacing of 130 km [18]. The Fireball Recovery and Interplanetary Observation Network (FRIPON) covers all of the French territory and employs 100 all-sky cameras with an average distance of 100 km between the stations [19]. In cooperation with the FRIPON, an initiative is also being developed, the Italian network PRISMA (Prima Rete per la Sorveglianza sistematica di Meteore e Atmosfera), intended to use gamma-ray spectrometers allowing one to reveal the presence of short-lived cosmogenic radioisotopes [20].

Utilization of a high frame rate camera results in a shorter exposure time, and it will naturally reduce the overall sensitivity of the imaging system. The earliest low-level television (LLTV) meteor observations were made in the 1960s with unintensified image orthicon cameras [21]. While this was a significant step forward in terms of sensitivity when compared with photographic techniques, the sensitivity was later improved by coupling with an image intensifier [22]. The image intensifiers are usually one of two basic types: (a) the first generation consisted of a photocathode followed by an accelerating electron lens, which focused the electron image onto the phosphor of the output window; (b) the second and third generation image intensifiers exploited the phenomenon of electron multiplication in a micro-channel plate (MCP). Several stages of first generation image intensifiers may be cascaded with a combined gain of the order of 100,000. Second generation intensifiers have gains approaching those of three-stage first generation device. In combination with fast and low noise CCD cameras, such a high-speed can detect stars down to about +8 to +9 apparent magnitude [23]. Recent development in CMOS technology suggests that MCP will be replaced by CMOS sensors in low-light-level (LLL) applications. Current CMOS sensors are reaching very low electron noise levels. Moreover, the quantum efficiency of CMOS-based devices can be up to 90% [24]. The state-of-the-art devices dedicated to low light level fast imaging are electron multiplying CCDs (EMCCDs), which are, however, still much more expensive in comparison to MCPs.

There are two main tasks for the software for meteor analysis: meteor detection (optionally in real-time) and determination of meteor parameters from double-station or network-based observations. This paper focuses on the first task, fast and reliable detection of meteors. The choice of a suitable method of detection strongly depends on the method of image acquisition, particularly on the frame rate of the image sensor. A typical meteor track is comprised of a streak lasting up to several video frames propagating linearly across space and time. For longer exposure times, typically used in all-sky systems, those streaks can be relatively long. Thus, a couple of works in this field employ the Hough transform [25,26].

Numerous papers deal with the problem of meteor streak detection. One of the most widely-used software applications performing the task of meteor detection TV systems, MetRec [27], works with the difference image to remove static stellar objects and calculates the sum of the pixels of potential neighboring objects in different directions. The object is considered to be a meteor if one or more of those sums exceeds a certain threshold. MeteorScan, a popular software package developed by Pete Gural [28], uses a technique based on frame differencing, variance-dependent thresholding and multi-frame Hough integration. Gural later proposed a method using matching filter [29], where an object’s motion is hypothesized for a particular starting point, speed and direction. Another popular package, UFOCapture (http://sonotaco.com/) applies a 5 × 5 spatial filter with frame differencing, which is then masked and thresholded. Kozak describes a typical meteor detection pipeline subtracting the average of 40–50 frames from the currently processed one [30]. CAMO employs All Sky and Guided Automatic Real-time Detection (ASGARD) [31]. CAMS and CMN use the method of detecting meteors in frames of digital video that had been compacted into a single bit-mapped color file [32]. Since this method produces large volumes of false detections (up to 50%), Silađi et al. [33] proposed a method using neural networks and support vector machine (SVM) to reach a 10% false rate. Molau and Gural [34] reported a real-time success-rate of meteor detection better than 80% for both MetRec and MeteorScan, with a false alarm rate less than 20%.

**Figure 1:**
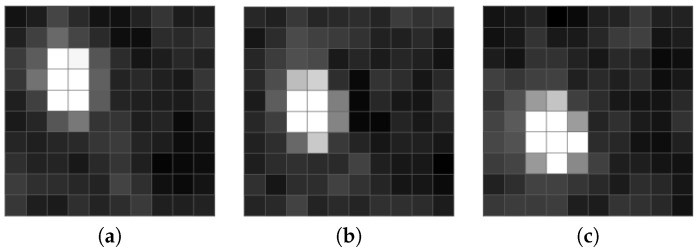
Detail of the sampled meteor streak. (**a**) Time T. (**b**) Time T + 30 ms. (**c**) Time T + 60 ms.

**Figure 2:**
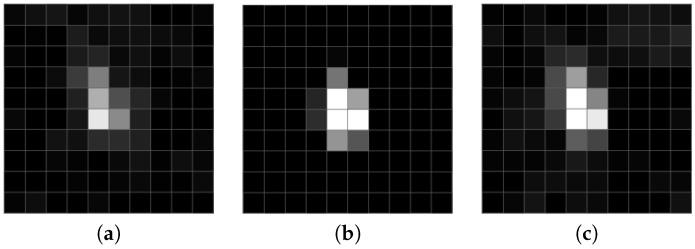
Detail of the static stellar object. (**a**) Time T. (**b**) Time T + 30 ms. (**c**) Time T + 60 ms.

**Figure 3:**
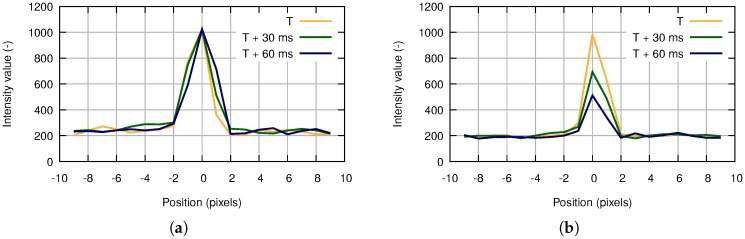
Profiles of the objects present in the frame. (**a**) Sampled meteor streak. (**b**) Stellar object.

All methods as mentioned above perform well while processing video sequences with less than VGA spatial resolution and a temporal resolution of no more than 25 fps. However, in the TV systems using frame rates of typically 25 fps or faster, meteor paths can be only a couple of pixels; see Figure 1 for example. As is shown in Figure 2, a meteor streak sampled at a high frame rate (here 61 fps) has a similar shape to stars. Figure 3 displays radial profiles of sampled meteor streaks in comparison with the sampled stellar object at the same times. Moreover, when an observing system employs non-linear devices like MPC, the algorithms have to deal with strong noise with a generally unknown distribution. Since we are targeting highly automated camera systems with minimal human interaction, our goal is also to minimize false alerts.

The paper is organized as follows. Section 2 introduces the characteristics of the second generation image intensifier. Section 3 describes a proposed algorithm for meteor detection in the video sequences acquired with the TV intensified system. Section 4 gives an overview of graphics processing unit (GPU)-based acceleration of our algorithm. Section 5 presents the results of real data processing, and, finally Section 6 concludes the paper.

## 2. Characterization of the MCP Image Intensifier

In this section, we will summarize the characteristics of the second generation MCP image intensifier. One of the representatives of this branch of imaging devices is Philips (Photonis) XX1332. The XX1332 image intensifier has a highly nonlinear input-output conversion function as a result of the automatic gain control (AGC). The AGC feature helps to accommodate extremely high dynamic range and also brings high nonlinearity, which is especially critical for photometric measurements. It calculates the mean intensity in the image and adjusts the multiplication gain (which results in higher excess noise) accordingly (increases if less photons are present and decreases for higher overall fluxes).

**Figure 4:**
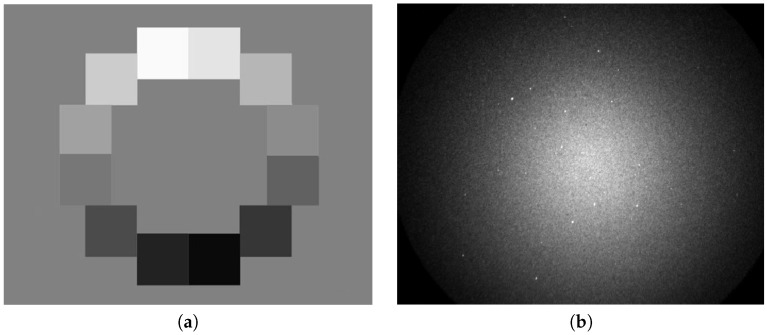
Examples of input data. (**a**) ISO 14524 test chart. (**b**) Real image data.

**Figure 5:**
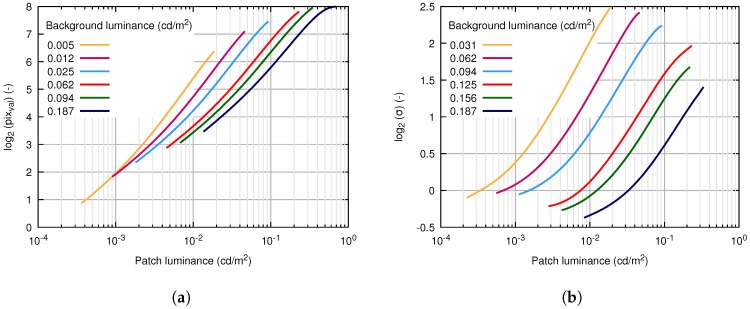
Image intensifier characteristics. (**a**) Opto-electronic conversion function dependence of the digital level in the output image on patch brightness for various background luminances. (**b**) Noise standard deviation dependence on patch brightness for various background luminances (gain levels of image intensifier). cd/m${}^{2}$: candela per square meter.

The AGC feature naturally affects the opto-electronic conversion function (OECF) of the instrument. To cover this characteristic, we used the ISO 14524 chart [35] (see Figure 4) illuminated under laboratory conditions. We used 17 various illuminance levels ranging between 1.6 mlx (mililuxes) and 2.4 lx, which leads to background luminance levels between 125 μcd/m${}^{2}$ (microcandela per square meter) and 187 mcd/m${}^{2}$ (milicandela per square meter). From the known illuminance and optical density of the particular patch, it is possible to calculate the patch luminance. Figure 5a displays OECF measured for six of 17 various background levels (gain levels in the image intensifier). It can be seen, however, that the OECF for the fixed gain is not perfectly linear; rather, high linearity is achieved.

The same ISO 15524 chart allows covering the dependency of noise characteristics on the spatially-variable signal level (represented by patches of the chart) and automatic gain control in the image intensifier. We also investigated the behavior of the image intensifier at different working conditions by the change of the chart illumination [36]. Figure 5b shows the curves for the six chosen illumination levels. Every curve represents the dependency of the standard deviation on the patch luminance. The several curves show that the system is highly signal dependent even in the case of constant illumination level (against the assumption, the standard deviation is growing with growing patch luminance). Furthermore, the standard deviation decreases with the growing background luminance.

The above-mentioned features, typical for the intensifier TV systems, cause the presence of speckle noise components in the acquired video sequences. The level of individual bright spots in the video frame fluctuates significantly, while the overall signal level remains roughly constant (i.e., a couple of bright spots increase their level, while the level is decreased for other bright spots). This phenomenon affects conventional image processing algorithms based on the subtraction concerning their scalability and performance. Together with findings from the measurement of the noise standard deviation, it naturally leads to the assumption that brighter parts (pixels) of the video sequence have higher variance.

**Figure 6:**
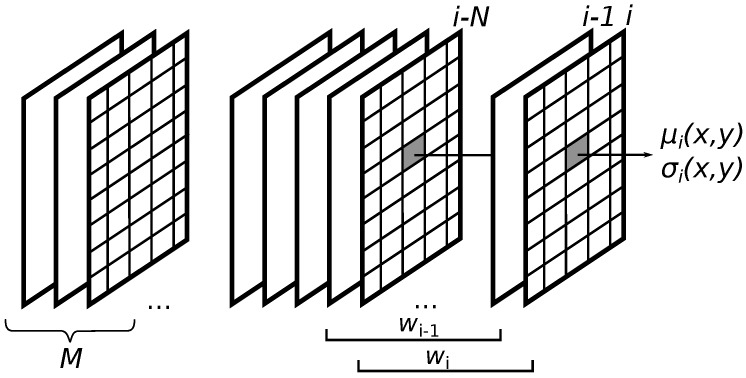
Idea of time-domain single pixel analysis.

This type of image acquisition system requires new methods of meteor detection. The idea arises from the previous analysis that it is difficult to examine the relationships between the pixels within one frame. We propose an algorithm that takes into account single pixel probability characteristics calculated across a certain number of frames. Figure 6 shows consecutive frames and the sliding window $w_{i}$ of size *N*. The value of the mean $\mu_{i}\left(x,y\right)$ and the standard deviation $\sigma_{i}\left(x,y\right)$ of the pixel at spatial position (x,y) in the *i*-th frame is calculated from the values of the pixel in the window. To detect a meteor, the algorithm searches for the relationship between pixel characteristics valid for the *i*-th frame and the model calculated from the first *M* frames of a video sequence.

**Figure 7:**
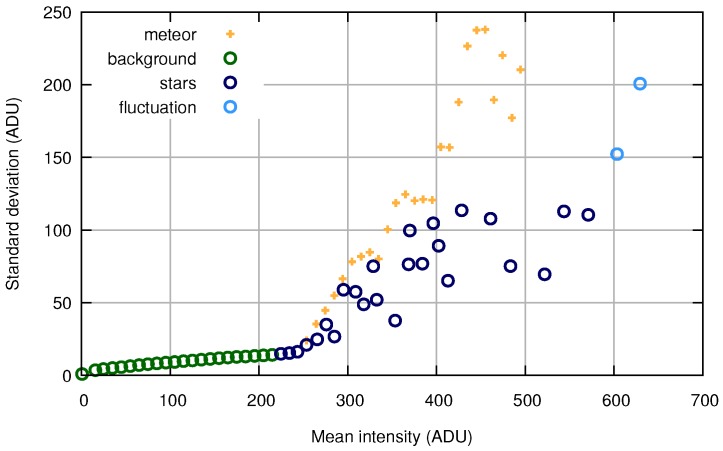
Comparison of statistical characteristics. ADU: analog-to-digital unit.

The model builds on the relation between the mean value and standard deviation of the pixels in the frame. In Figure 7, the circle marks present this relation in frames without a meteor, and it demonstrates an example of how this relation changes when a sliding window includes frames with a meteor. One can see a deviation in a certain interval of pixel intensity values caused by the temporal appearance of the meteor on the dark background, which increases the standard deviation of pixels with low intensity.

Both, meteor appearance and the noise can increase the mean and standard deviation of a pixel across a window. Thus, it is not enough to keep these parameters for each pixel. The model has to describe the estimation of the permitted standard deviation depending on the mean intensity. In this case, we propose to construct the model by approximation of the relation between the mean value and the standard deviation in frames without a meteor. We consider video sequences of a duration of 10 min and propose the renewal of the model from the last *M* frames labeled as frames without a meteor. It compensates variations during the night.

## 3. Description of the Algorithm

**Figure 8:**
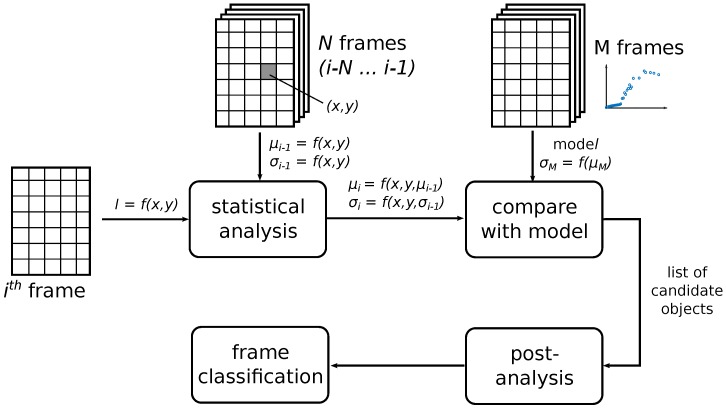
Block diagram of the proposed classifier.

Based on the above-described idea, we propose the frame classification method shown in Figure 8. The statistical analysis block provides the calculation of the mean value $\mu_{i}\left(x,y\right)$ and standard deviation $\sigma_{i}\left(x,y\right)$ of each pixel through *N* frames. We use the recursive calculation of this characteristic based on known $\mu_{i-1}\left(x,y\right)$ and $\sigma_{i-1}\left(x,y\right)$. With the model built from the frames with only static objects present, an algorithm can detect the transient (i.e., moving) object. To reduce false detection, we also introduce the post-analysis block exploring how many times the algorithm marked the single pixel and its neighbors as candidate objects.

### 3.1. Statistical Analysis

Widely-used methods of computing the standard deviation require two passes through the data. Since our effort focuses on real-time data processing, more suitable for implementation are single-pass algorithms. Our pipeline uses the robust iterative formula by Welford [37]. Since we have determined the mean $\mu_{0}$ and variance $\sigma_{0}^{2}$ of a single pixel for the window included in (*i*, i+1, ... i+N−1) frames, we can estimate how parameters $\mu_{1}$ and $\sigma_{1}^{2}$ change when we slide the window by one position:

(1) $$\mu_{1}-\mu_{0}=\frac{\sum_{i=1}^{N}I_{i}-\sum_{i=0}^{N-1}I_{i}}{N}=\frac{I_{N}-I_{0}}{N},$$

where $I_{i}$ is the pixel intensity in the *i*-th frame and *N* is the window size. To evaluate the difference of variances, the unbiased sample variance is used:

(2) $$\sigma^{2}=\frac{\sum_{i=1}^{N}I_{i}^{2}}{N-1}-\frac{{\left(\sum_{i=1}^{N}I_{i}\right)}^{2}}{(N-1)N}.$$

Hence, we obtain:

(3) $$\begin{array}{c} \left(N-1\right)\sigma_{1}^{2}-\left(N-1\right)\sigma_{0}^{2}=\left(\sum_{i=1}^{N}I_{i}^{2}-N\mu_{1}^{2}\right)-\left(\sum_{i=0}^{N-1}I_{i}^{2}-N\mu_{0}^{2}\right)\\ =I_{N}^{2}-I_{0}^{2}-N\left(\mu_{1}^{2}-\mu_{0}^{2}\right)=\left(I_{N}-I_{0}\right)\left(I_{N}-\mu_{1}+I_{0}-\mu_{0}\right). \end{array}$$

This means that we can use the iteration formula to calculate the mean value and the variation of a pixel across the window of size *N* frames. In our algorithm, we use a window size equal to 15 frames, which is enough to follow the changes of the standard deviation and to detect a meteor in a frame.

### 3.2. Comparison with the Model

To get the list of candidate objects in the *i*-th frame, we perform the statistical analysis across the moving window of size *N*. The calculated standard deviation of a single pixel at spatial position (x,y) with a certain mean value is compared with a corresponding value of the a priori model $\sigma_{M}=f\left(\mu_{M}\right)$. If the standard deviation of the pixel is significantly higher than the model standard deviation, we label this pixel as a candidate object.

The model represents the relationship between the mean of pixel values $\mu_{M}$ and the standard deviation $\sigma_{M}$, and it is constructed from data samples of *M* frames including static objects only (typically the first 15 frames in a video sequence). To get these data samples, we calculate the mean values and standard deviation of each pixel across *M* frames. In this case, the number of samples associated with the background is significantly bigger. To get an equal number of samples in different intervals of dynamic range, we average these parameters in single intervals.

**Figure 9:**
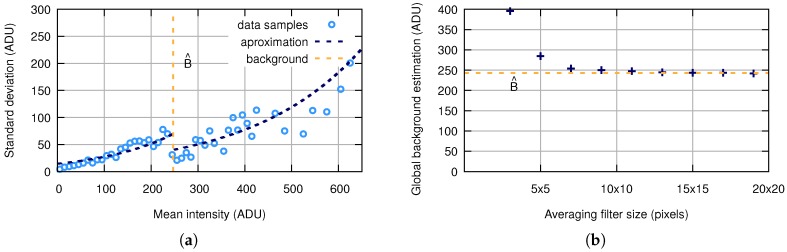
(**a**) The model of the statistical characteristic dependency. (**b**) Size of the averaging filter for the global background estimation.

The precision of the model is a crucial factor affecting algorithm performance. We found that the model is well described by the formula:

(4) $$\sigma_{M}=\left\{\begin{array}{cc} a_{1}\cdot e^{b_{1}\mu_{M}}, & \mu_{M}\leq \hat{B};\\ a_{2}\cdot e^{b_{2}\mu_{M}}, & \mu_{M}>\hat{B}, \end{array}\right.$$

where $\hat{B}$ is an estimation of the background and $a_{1,2}$ and $b_{1,2}$ are parameters approximating data samples from *M* frames. Accurate background estimation significantly reduces the number of detection errors. There are different methods, for example sigma-clipping [38], multiresolution support [39], modified distance transform [40], etc. The trade-off between efficiency, simplicity and speed leads to the use of the convolution with the averaging filter [41] of size 11×11 pixels for this particular task. An example of how the model fits the data samples can be seen in Figure 9a. Figure 9b shows the dependency of the estimated background value on the size of the filter.

### 3.3. Post-Analysis

**Figure 10:**
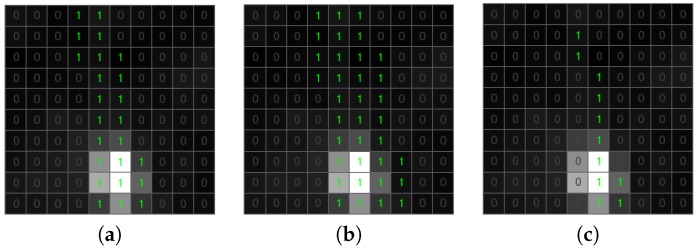
Morphological transformation of a positive candidate object. (**a**) Candidate object. (**b**) Dilation. (**c**) Erosion.

**Figure 11:**
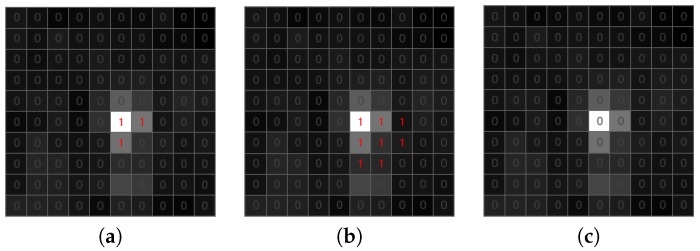
Morphological transformation of a negative candidate object. (**a**) Candidate object. (**b**) Dilation. (**c**) Erosion.

The list of candidate objects includes both true and false positive detections. False candidate objects are typically one-pixel or a small connected area; most of them can be removed efficiently by the use of the morphological transformation [42]. In the proposed algorithm, we apply dilation followed by erosion (Figure 10 and Figure 11). Dilation with 2×2 structuring element allows connecting candidates that are close to each other and ensuring saving a meteorite trajectory following erosion (Figure 10). Erosion with 3×3 structuring element removes all candidates that have no eight-connectivity, which is an effective way to get out of negative candidates (Figure 11). Using bigger structuring elements for morphological transformation can cause the removal of the meteor trajectory.

**Figure 12:**
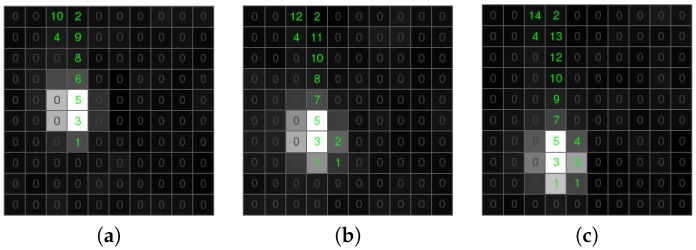
The counters of a positive candidate associated with pixels labeled as a candidate object. (**a**) Time T. (**b**) Time T + 30 ms. (**c**) Time T + 60 ms.

**Figure 13:**
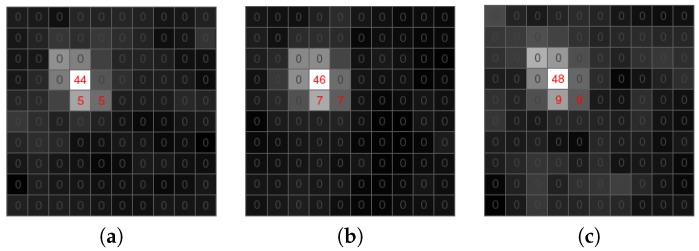
The counters of a negative candidate associated with pixels labeled as a candidate object. (**a**) Time T. (**b**) Time T + 30 ms. (**c**) Time T + 60 ms.

Classification of residual candidate objects requires further analysis. Our algorithm uses a counter calculating how many times the single pixel was marked as a candidate object in previous frames. We analyzed the results of this calculation for a meteor and a static object. In Figure 12, the green path is a meteor trajectory. The counter associated with the positive candidate objects tends to decrease its value in the direction of an object moving smoothly. As we can see, the counters of single pixels have no big difference from their non-zero neighbors. This allows excluding candidates having significant differences in counters associated with the negative candidate (Figure 13). Based on this assumption, we define the difference between a pixel’s counter and its non-zero neighbors. If the biggest difference is lower than four, we mark a pixel as an object.

The result of post-analysis is a list of detected meteors in the frame, which is the basis for frame classification. If the list is not empty, we mark the frame as including a meteor.

## 4. GPU Acceleration

**Figure 14:**
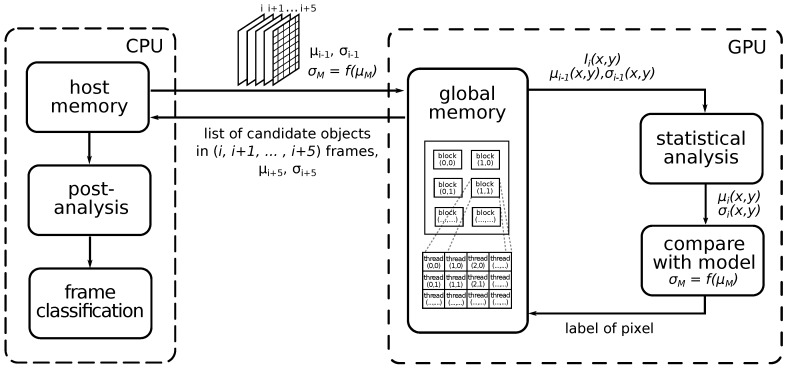
Block diagram of the GPU-accelerated pipeline.

Besides true positive detections of meteors, the second most important parameter of the algorithm is an execution time. The algorithm was designed to be implemented on a GPU using CUDA (Compute Unified Device Architecture), a highly parallel multi-threaded architecture [43]. A block diagram of this implementation is shown in Figure 14. One of the main bottlenecks of GPU acceleration is inefficient data transfer between the host and the device, negatively affecting the overall application performance. Thus, our GPU implementation simultaneously processes several frames, as proposed by Vítek in [44]. We transfer frames $\left(i,i+1,\ldots ,i+N_{gpu}-1\right)$ to the GPU global memory where $\left(i=1,\;N_{gpu}+1,\;2\cdot N_{gpu}+1,\ldots \right)$, and $N_{gpu}$ is the window size chosen based on the parameters of the GPU. After some experiment, we found that six-frame processes simultaneously represent a good-enough trade-off between accuracy and execution time.

The recursive calculation of the statistic characteristics described in Section 3.1 is used for all transferred frames except the last one. To get ($\mu_{i},\mu_{i+1},\ldots ,\mu_{i+N_{gpu}-2})$ and ($\sigma_{i},\sigma_{i+1},\ldots ,\sigma_{i+N_{gpu}-2})$, only one set of referent parameters $\mu_{i-1}$ and $\sigma_{i-1}$ is used. This is the main difference from the CPU implementation, which uses referent parameters for each frame. The statistic characteristics of the last frame in the window have to be calculated based on all frames in the window without using recursion because it defines the accuracy of detection in the next frames.

## 5. Verification of Algorithm Performance

**Figure 15:**
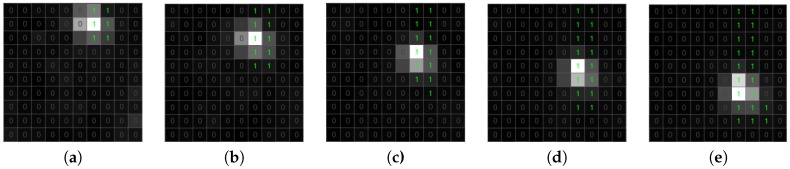
True positive example. (**a**) Time T. (**b**) Time T + 15 ms. (**c**) Time T + 30 ms. (**d**) Time T + 45 ms. (**e**) Time T + 60 ms.

**Figure 16:**
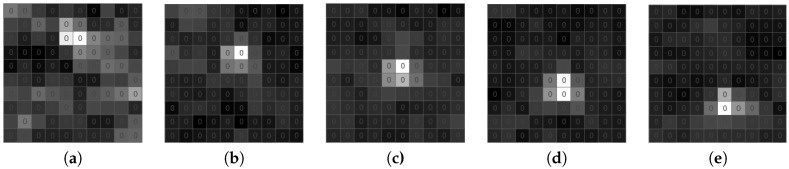
False negative example. (**a**) Time T. (**b**) Time T + 15 ms. (**c**) Time T + 30 ms. (**d**) Time T + 45 ms. (**e**) Time T + 60 ms.

During the test, we focused on the two main features of the algorithms: (a) the ability to detect meteors in the single frame and (b) the ability to detect an event as such. The frame classifier has four possible outcomes: true positive, shown in Figure 15 (TP, the case when the meteor is present in the frame and it is correctly detected by the algorithm), false positive (FP, the case when the meteor is not present in the frame, but it is falsely detected by the algorithm), true negative (TN, the case when the meteor is not present in the frame and the algorithm is not producing any alert) and false negative, shown in Figure 16 (FN, the case when the meteor is present in the frame and the algorithm is not producing any alert).

Performance of the detection algorithm depends on the geocentric velocity of the meteoroids and the geometry of the meteor appearance. When a meteoroid enters the top layers of the Earth’s atmosphere, its movement is not followed by any significant change in brightness. Thus, it is hard to distinguish the beginning of the event and fluctuation caused by speckle noise, and frames capturing the beginning of the meteor trail are the main source of the FN classification. Another problem for the processing algorithm is faint meteors, for example meteoroids entering the atmosphere at a small angle, so the overall duration of the event is short, and changes in the brightness are weak. It is therefore difficult to track the brightness changes of the neighboring pixels, and the false detection rate is higher for frames capturing shorter events.

### 5.1. Experimental Setup

For the purpose of this paper, testing data were acquired with the system MAIA (Meteor Automatic Imager and Analyzer) [45]. This system uses image intensifier XX1332 and GigE (Gigabit Ethernet) progressive scanning camera JAI CM-040GE, running at a frame rate of 61 fps and a bit depth of 10 bits. The spatial resolution of the camera is 776 × 582 pixels (approximately 6 arcmin/pixel), corresponding to a field-of-view of 52${}^{\circ }$. The limiting stellar magnitude is +8. The astrometric precision of the system is quite good: the standard deviation is better than 0.04${}^{\circ }$ both for naked and intensified systems. MAIA works in double-station configuration, and camera systems are deployed in two places: Ondřejov and Kunžak, the distance between both stations being 92.5 km.

To evaluate the performance of the proposed algorithm, we processed 30 video sequences with a total number of 2419 frames, acquired during different nights by the use of the MAIA system. All video sequences contain a meteor, and we manually labeled all 1169 frames on which meteors are recorded. Frames at the beginning of each video sequence contain only static objects, so it is possible to build the model. We compared our algorithm with three other methods: the first one is an algorithm that is currently in use within the MAIA project; the second one is the widely-used UFOCapture; and the third one is our reimplementation of a meteor detector used within CMN [46]. Originally, the Python-based software RPi Meteor Station (RMS [47]) was running on the Raspberry Pi platform.

The algorithm currently in use within the MAIA project takes into account the high temporal resolution of video sequences. It creates a list of static objects and detects new objects in the next frames. Each new object is placed in the list of temporary objects as an object for the next investigation. To find a meteor, the trajectories of these temporary objects are followed. The algorithm is implemented in the pipeline known as dMAIA. The goal of the pipeline is obtaining the sequential measurement of the meteor and its apparent coordinates in comparison with real stars in the background. Detected meteors are the subject of further measurements, particularly the measurement of brightness, the measurement of range of height (especially the beginning heights) and the determination of the atmospheric trajectory. Details about the measurement may be found for example in [48,49].

The most common methods of meteor detection discover meteor tracks in video sequences with low temporal resolution. In this case, the meteor track presents a line in each frame. The RMS algorithm is based on this frame feature. Its basic concept is line detection by kernel-based Hough transform in a reconstruction image from 64 frames. In our implementation of this algorithm, we reconstructed images from 15 frames, which was enough to detect a meteor.

### 5.2. Results

To compare the execution time of a tested algorithm, we used a personal computer with Intel Core i5-3210M 2.5 GHz x4, 16 GB of DDR3/1600 MHz memory and NVIDIA GeForce GT 635M 2 GB GDDR5 graphics card. To include UFOCapture in the test, we developed a custom virtual DirectShow camera. As we can see in Table 1, the implementation of the proposed algorithm significantly reduces the time needed to process one frame of the video sequence. Note that the time needed to build a model is 1.19 s, so while we are updating the model once per 36,600 frames (i.e., ten minutes of recording), there is an overhead of 0.03 ms per processed frame.

Table 2 summarizes the results of particular algorithms. Following our hypothesis of the more difficult detection of shorter events, we performed a test on the subset of video sequences containing events longer than 25 frames. The results of those tests are summarized in Table 2b, and one can see a significantly lower number of FN detections for events longer than 25 frames.

Furthermore, we evaluated the ability of the algorithms to find a meteor event (i.e., a streak of light in consecutive frames) in the video sequence. Our algorithm was able to detect all meteors in the video sequences, and the currently used algorithm missed two meteors, while the algorithm based on RMS missed three meteors. UFOCapture missed only one meteor, but also produced a high number of false positives.

It is worth noting that we also had the possibility to investigate the usability of the tracking algorithm incorporated in a University of Western Ontario processing pipeline for high temporal resolution of video sequences. This algorithm is an evolution of the Astrobiology Instrumentation for Meteor Imaging and Tracking system [50]. It has an advantage in time processing compared with the proposed algorithm. The time of a single frame processing is 6.3 ms. However, this algorithm requires the accurate setting of input parameters for each video sequence, which has a significant effect on the precision of meteor detection. The algorithm proposed in this paper removes this disadvantage.

## 6. Conclusions

This paper focuses on methods of meteor detection in video sequences with high frame rates. We proposed the algorithm of frame classification based on the comparison between temporal statistical characteristics of a pixel and the model built on the relation between the mean and the standard deviation of the pixel.

The results showed high performance in accuracy and speed. The precision of the proposed algorithm is 0.9881, and the recall is 0.8503. The proposed algorithm is significantly faster compared to state-of-the-art algorithms. The implementation using computing on a GPU reduced the processing time of a single frame and had a duration of 10.3 ms per frame, which means that it is possible to process single frame in a time shorter than the exposure time (16.4 ms for a frame rate of 61 fps). The parameters of this implementation need further investigation to obtain a trade-off between accuracy and speed.

References

## Figures and Tables

**Table 1:** Time for processing a single frame.

	CPU (ms)	GPU (ms)
UFOCapture	62.6	-
RMS	47.1	-
dMAIA	27.2	16.1
Proposed	12.2	10.3

**Table 2:** Confusion matrix based on frame classification. (a) Analysis of all available video sequences. (b) Analysis of the video sequences capturing events longer than 25 frames.

	(a)				(b)			
	TP	FP	FN	TN	TP	FP	FN	TN
UFOCapture	920	320	249	950	425	93	81	274
	78.69%	25.19%	21.31%	74.81%	83.99%	25.34%	16.01%	74.66%
RMS	860	14	309	1256	391	**0**	115	**367**
	73.57%	1.1%	26.43%	98.9%	77.27%	**0%**	22.73%	**100%**
dMAIA	902	22	267	1248	420	10	86	357
	77.15%	1.73%	22.84%	98.26%	83%	2.72%	17%	97.18%
proposed	**994**	**12**	**175**	**1258**	**462**	5	**44**	362
	**85.03%**	**0.94%**	**14.97%**	**99.06%**	**91.03%**	1.36%	**8.07%**	98.64%
